# Broadband Dielectric Spectroscopy with a Microwave Ablation Antenna

**DOI:** 10.3390/s23052579

**Published:** 2023-02-26

**Authors:** Klementina Vidjak, Carolin Hessinger, Marta Cavagnaro

**Affiliations:** 1 Department of Information Engineering, Electronics, and Telecommunications, Sapienza University, Piazzale Aldo Moro 5, 00185 Rome, Italy; 2 Institute for Microwave Engineering and Photonics, Technische Universität Darmstadt, Merckstr. 25, 64283 Darmstadt, Germany

**Keywords:** microwave ablation treatment, microwave ablation antenna, dielectric spectroscopy

## Abstract

Microwave ablation is a technique used to treat tumorous tissue. Its clinical use has been greatly expanding in the last few years. Because the design of the ablation antenna and the success of the treatment greatly depend on the accurate knowledge of the dielectric properties of the tissue being treated, it is highly valuable to have a microwave ablation antenna that is also able to perform in-situ dielectric spectroscopy. In this work, an open-ended coaxial slot ablation antenna design operating at 5.8 GHz is adopted from previous work, and its sensing abilities and limitations are investigated in respect of the dimensions of the material under test. Numerical simulations were performed to investigate the functionality of the floating sleeve of the antenna and to find the optimal de-embedding model and calibration option for obtaining accurate dielectric properties of the area of interest. Results show that, as in the case of the open-ended coaxial probe, the accuracy of the measurement greatly depends on the likeness between the calibration standards’ dielectric properties and the material under test. Finally, the results of this paper clarify to which extent the antenna can be used to measure dielectric properties and paves the way to future improvements and the introduction of this functionality into microwave thermal ablation treatments.

## 1. Introduction

Electromagnetic fields are utilized in medicine for therapeutical techniques such as in radiofrequency ablation (RFA) [1,2], microwave thermal ablation (MWA) [3,4], and hyperthermia (HT) [5]. While these techniques have been used more extensively in recent years, a lot still needs to be investigated and improved. 

MWA technique uses antennas operating in the microwave frequency range to destroy unhealthy tissue. During treatments, the tissue is heated to temperatures around 60 °C, which causes cellular necrosis [6]. The technique is eligible for treating different tumors, such as liver tumors, lung tumors, renal tumors, and bone tumors. To design an appropriate MWA antenna, accurate knowledge of the dielectric properties of the targeted tissue is required. Many different antenna designs, including monopoles, dipoles, and coaxial slot antennas, are found in the literature [7]. The optimal MWA antenna design is a tradeoff of different ablation parameters such as matching of the antenna, sphericity, size of the thermally ablated zone, and invasiveness of the treatment in the sense of antenna diameter and length. 

Still, the appropriate antenna design does not guarantee a successful treatment. Currently, ablation antennas are being guided into the tumor using ultrasound which is unable to give any information upon the start of ablation due to the formation of water vapor, which blinds the ultrasound probe [8]. Another option for monitoring the condition of the tissue around the antenna during the procedure is with computerized tomography (CT), but this is quite costly, uses ionizing radiation, and causes difficulties in the ablation procedure [9]. In this respect, the knowledge in real-time of the dielectric properties of the tissue in which the antenna is immersed could indicate whether the tissue is healthy or malignant or if the procedure was performed correctly. In fact, tumors, as well as thermally ablated tissue, show different dielectric properties with respect to healthy tissues [3,10]. Therefore, enabling the ablation antenna to both monitor the condition of the tissue (measure dielectric properties) and ablate is crucial. So far, there have been some attempts at creating such microwave applicators [11,12,13], but further studies are required to implement this expanded MWA technique in operating theaters.

In this work, a dual-mode open-ended coaxial slot antenna was investigated [12,13]. Depending on the mode, this antenna can perform both microwave thermal ablation (MWA) and sensing. Unlike commonly used MWA antennas operating at either 915 MHz or 2.45 GHz, this one operates at 5.8 GHz. The higher frequency allows a smaller antenna design and ensures more spherical ablation zones in the tissue enabling the ablation of very small tumors [14]. While operating in sensing mode, the antenna measures the dielectric properties of the surrounding tissue by means of the reflection coefficient. 

In previous works [12,13], the sensing capabilities of the antenna were verified at the antenna operating frequency only and with the antenna inserted in a liver-filled simulation block with fixed dimensions of 100 × 100 × 100 mm. In this research, the antenna sensing ability is tested on a broad frequency range both numerically and experimentally. To this end, different de-embedding options are investigated and compared. Furthermore, a numerical study of the sensitivity of the antenna to the transversal and longitudinal dimensions of the material under test (MUT) is reported. 

## 2. Materials and Methods

In this section, the antenna design, as well as the software used for the simulations, are explained. Then, the de-embedding models used for determining dielectric properties of MUT are presented along with the definition of antenna sensitivity. Finally, the dielectric properties of all used materials are given. 

### 2.1. Antenna Model and Numerical Simulation Settings

**Figure 1:**
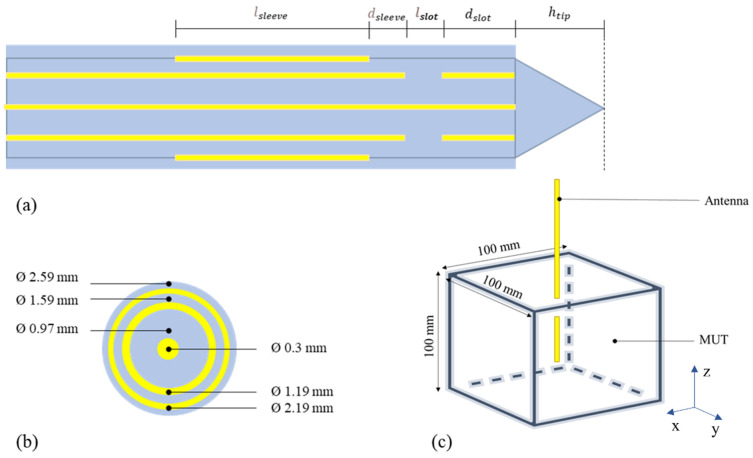
(**a**) Antenna design transversal view; (**b**) Cross section of the coaxial cable used for the design; (**c**) Geometry of the simulations. Metal parts are represented in yellow, while the insulation materials are represented in blue.

The antenna was designed for application in liver tissue, and, therefore, it needed to resonate at 5.8 GHz in such a dielectric ambient [15]. It is a coaxial slot antenna with an open end. A floating sleeve is introduced to ensure efficient energy absorption into the surrounding tissue and avert reverse currents on the outside of the antenna [16]. The general geometry of this applicator is shown in Figure 1. 

To find the best dimensions of geometrical parameters of the applicator to optimize the antenna’s efficiency (reflection coefficient below −10 dB) and ablation zone size and shape at the operating frequency, the Pareto-optimization method was used [17]. The optimization process gave several Pareto-optimal parameters sets, all with very similar objective values. Aiming to study the sensing mode of the antenna, the geometry replicated in the simulations of this work was the one that, along with good size and sphericity of the ablation zone, achieved the best sensitivity in the measurement of the dielectric properties of the MUT (2.04%) while maintaining good matching at 5.8 GHz (−18.61 dB).

Following the description of the antenna design and the simulation settings from [13], the antenna was implemented in CST Studio Suite^®^ 2021 (Dassault Systèmes, Vélizy-Villacoublay, France). Table 1 gives an overview of all the dimensional parameters used in the simulation. The metal parts of the antenna were simulated as perfect electric conductors (PEC), and the dielectric was simulated with lossless PTFE with permittivity 2.1. The transversal view of the antenna and the cross-section of the coaxial cable are shown in Figure 1a,b, respectively.

The simulations were performed using the transient solver of CST, in the frequency range 0–10 GHz with 1001 linearly spaced points. The antenna was immersed for 50 mm in a cubic-shaped liver-filled MUT with dimensions of 100 × 100 × 100 mm (Figure 1c). The boundary conditions were set to “open (add space),” which means that at least 1 additional wavelength of space (at 5 GHz; center frequency of the simulation) is added around the MUT and the antenna, with an estimated reflection level at the boundary of 0.0001. The MUT and the antenna shaft were backed by air. To reduce the computational time, two symmetry planes were used. To be more specific, the antenna was placed along the z-axis, and YZ and XZ turned out to be symmetry planes because the magnetic field is perpendicular to them (has no tangential component; H_t_ = 0). Accordingly, the magnetic symmetry condition of CST was selected.

The proposed antenna was fabricated from the semirigid coaxial cable of type UT085, with an outer diameter of 2.1 mm. The reflection coefficient of the antenna prototype immersed in reference materials, namely distilled water (DW), 0.1 mol, and 2 mol sodium chloride (NaCl) solutions and left in the air (OC condition), was measured to verify the antenna design and validate the numerical model. 

**Figure 2:**
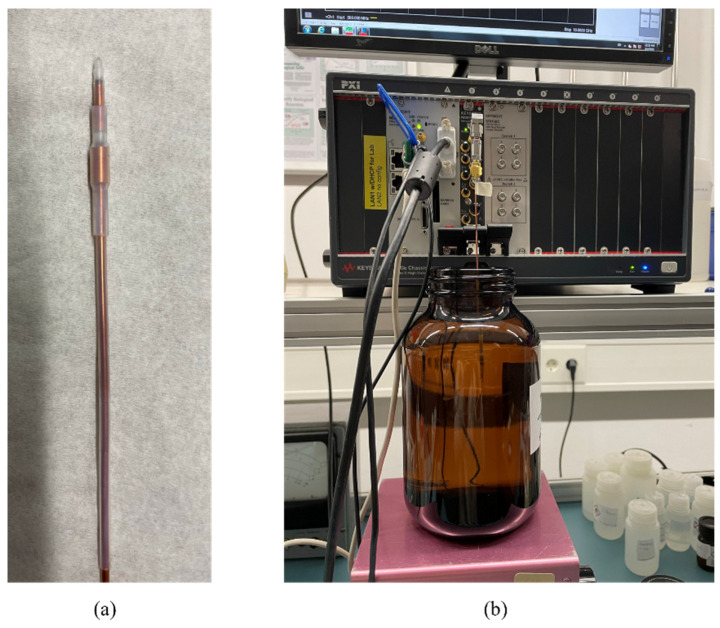
(**a**) Realization of the applicator; (**b**) measurement setup with the antenna immersed into the liquid-filled tank.

The reflection coefficient measurements were performed using a vector network analyzer (VNA; PXI M9375A, Keysight Technologies, Santa Rosa, California, the US). Figure 2 shows the physical realization of the antenna applicator (a) and the measurement setup (b). The measurements were performed in a glass container with the antenna placed in the center.

### 2.2. De-Embedding Models for Reconstructing Dielectric Properties

In the original work [12,13], to de-embed dielectric properties of the liver from its simulated/measured reflection coefficient, the Stuchly & Stuchly model (S&S) was used [18]. The S&S model uses an equivalent circuit of the probe tip made by capacitances, in which the dielectric properties of the MUT are embedded [18]. This model is designed and used for the open-ended coaxial probe technique for measuring dielectric properties [18]. Nonetheless, it performed well with this antenna when used to measure dielectric properties at its operating frequency [13]. 

Additionally, in this work, another de-embedding model, still based on an equivalent circuit, was used. The Marsland & Evans model (M&E) is also intended for the open-ended coaxial probe [19] but with respect to the S&S circuit, it includes an additional element representing the radiation conductance. Being the sensing probe, in this case, an antenna, the M&E model is, therefore, expected to give more precise results than the S&S one.

**Figure 3:**
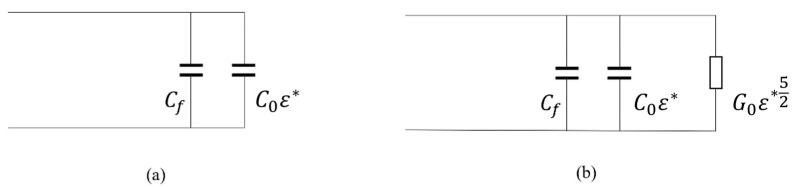
(**a**) Equivalent circuit of the S&S de-embedding model; (**b**) Equivalent circuit of the M&E de-embedding model.

The two circuits used by S&S and M&E are shown in Figure 3 where:

- capacitance $C_{f}$ represents the fringing field in the dielectric core of the probe; it is the capacitance between the inner and outer conductor of the probe,
- capacitance $C_{0}\varepsilon^{*}$ represents the fringing field in the outer dielectric material (MUT); capacitance $C_{0}$ represents the fringing field in free space (when no MUT is located at the probe’s aperture),
- conductance $G_{0}\varepsilon^{*}{}^{\frac{5}{2}}$ represents the radiation conductance of the probe; conductance $G_{0}$ is the radiation conductance in free space.

To express the relationship between probe input admittance and dielectric permittivity of the MUT, Deschamps’s antenna modeling theorem is applied [20]. It is applicable to any probe geometry under the condition that the surrounding medium is infinite, meaning the radiation field must be completed within the medium. When the medium is non-magnetic (μ_r = 1), Deschamps’s theorem can be written as [20]:

(1) $$Y(\omega ,\varepsilon^{*})=\sqrt{\varepsilon^{*}}Y\left(\sqrt{\varepsilon^{*}}\omega ,\varepsilon_{0}\right),$$

where ε* is the complex permittivity of MUT, *ω* is the angular frequency, *ε*_0_ is the permittivity of vacuum, and *Y* is the antenna admittance.

Equation (1), in combination with Marcuvitz’s analytic expression for the equivalent circuit admittance of a coaxial line open into free space [21] enables the writing of the expressions for the probe load admittance. The probe load admittance, normalized to the characteristic impedance of the probe’s fundamental mode Z_0_, takes the following form [22]:

- S&S model

(2) $$y(\omega ,\varepsilon^{*})=j\omega Z_{0}(C_{0}\varepsilon^{*}+C_{f})$$

- M&E model

(3) $$y(\omega ,\varepsilon^{*})=Z_{0}G_{0}\varepsilon^{*}{}^{\frac{5}{2}}+j\omega Z_{0}(C_{0}\varepsilon^{*}+C_{f}),$$

The power 5/2 in Equation (3) originates from the fact that the radiation conductance is proportional to the fourth power of frequency. When the coaxial line dimensions are small in comparison to the wavelength, then *G*_0_ ≪ *ωC*_0_, leading to a simplification of the equivalent circuit in which the radiation conductance is completely disregarded. In this case, Equation (2) reduces to Equation (1).

To compute the permittivity from the reflection coefficient, obtained from either simulation or physical measurements, using Equations (3) or (4), the relationship between admittance and true reflection coefficient, Γ_m_, and the relationship between measured reflection coefficient, $\rho_{m}$, and admittance are used. After some mathematical manipulations, final expressions for computing the measured permittivity are obtained, dependent on the permittivity of known liquids used as calibration standards [22]:

- S&S model

(4) $$\varepsilon^{*}=-\left[\frac{\Delta_{32}\Delta_{m1}y_{1}^{\prime }y_{3}^{\prime }+\Delta_{21}\Delta_{m3}y_{1}^{\prime }y_{2}^{\prime }+\Delta_{13}\Delta_{m2}y_{2}^{\prime }y_{3}^{\prime }}{\Delta_{21}\Delta_{m3}y_{3}^{\prime }+\Delta_{32}\Delta_{m1}y_{2}^{\prime }+\Delta_{13}\Delta_{m2}y_{1}^{\prime }}\right],$$

- M&E model

(5) $$G_{n}\varepsilon^{*}{}^{5/2}+\varepsilon^{*}+\left[\frac{\Delta_{32}\Delta_{m1}y_{1}^{\prime }y_{3}^{\prime }+\Delta_{21}\Delta_{m3}y_{1}^{\prime }y_{2}^{\prime }+\Delta_{13}\Delta_{m2}y_{2}^{\prime }y_{3}^{\prime }}{\Delta_{21}\Delta_{m3}y_{3}^{\prime }+\Delta_{32}\Delta_{m1}y_{2}^{\prime }+\Delta_{13}\Delta_{m2}y_{1}^{\prime }}\right]=0,$$

where:

- $G_{n}=G_{0}/j\omega C_{0}$ is the normalized radiation conductance,
- $\Delta_{ij}=\rho_{i}-\rho_{j}$ is the difference in reflection coefficients, *ρ_i.j_* is the measured reflection coefficient when the probe is immersed in one of the known calibration standards (*i, j* = 1, 2, 3), *ρ_m_* is the measured reflection coefficient when the probe is immersed in the MUT,
- $y_{j}^{\prime }$ is the input probe admittance when it is immersed in one of the known calibration standards (*j* = 1, 2, 3).

As derived from Equations (4) and (5), the S&S model requires three calibration standards, while for calculations with M&E, four are needed. In this work, these include open-circuit (OC) and liquids with well-characterized dielectric properties. Equations (4) and (5) were used to calculate the complex permittivity at each frequency of interest.

To evaluate the performance of the antenna in measuring the dielectric properties of the MUT, the sensitivity is defined. Sensitivity is expressed as a percentage deviation of the calculated dielectric properties from the reference properties [23]:

(6) $$\Delta_{real}=\frac{\left|\varepsilon_{ref}^{\prime }-\varepsilon_{res}^{\prime }\right|}{\varepsilon_{ref}^{\prime }}\times 100,$$

(7) $$\Delta_{imag}=\frac{\left|\varepsilon_{ref}^{\prime \prime }-\varepsilon_{res}^{\prime \prime }\right|}{\varepsilon_{ref}^{\prime \prime }}\times 100,$$

where:

- $\varepsilon_{ref}^{\prime }$ and $\varepsilon_{ref}^{\prime \prime }$ are the real and imaginary parts of reference complex permittivity of the MUT at the given frequency,
- $\varepsilon_{res}^{\prime }$ and $\varepsilon_{res}^{\prime \prime }$ are the real and imaginary parts of the calculated complex permittivity, obtained using a specific de-embedding model and a combination of calibration standards.

#### Dielectric Properties of Selected Materials

As stated in Section 2.2, to be able to perform dielectric measurements, a calibration step is required. In the calibration step, the antenna is inserted in materials with known dielectric properties. In this work, to look for the best combination of calibration standards, different materials were simulated. Liver, described in [15], was used as the MUT, while distilled water (DW) [24], three sodium chloride solutions (0.1, 1, and 2 mol NaCl) [25] and a 70% mixture of ethylene glycol and distilled water (EG70) [26] were used as reference materials in the de-embedding of MUT’s properties. 

The dielectric properties of the liver are described with a 1-pole Cole–Cole model [27]:

(8) $$\varepsilon^{*}=\varepsilon_{\infty }+\frac{\varepsilon_{s}-\varepsilon_{\infty }}{1+{\left(j\omega \tau \right)}^{1-\alpha }}+\frac{\sigma_{i}}{j\omega \varepsilon_{0}}=\varepsilon^{\prime }-j\varepsilon^{\prime \prime },$$

where:

- *ε_∞_* is the infinite permittivity (at a very high frequency),
- *ε_s_* is the static permittivity,
- *τ* is the relaxation time constant,
- *ω* is the angular frequency,
- *α* is an empirical parameter for broadening the dispersion,
- *σ_i_* is the ionic conductivity,
- *ε*_0_ is the permittivity of the vacuum.

The value of parameter α in Equation (8) for the liver is equal to 0.1 [15]. When α is equal to 0, Equation (8) takes the form of the Debye model [28]. This model is used to describe the dielectric properties of water and sodium chloride solutions used in this study:

(9) $$\varepsilon^{*}=\varepsilon_{\infty }+\frac{\varepsilon_{s}-\varepsilon_{\infty }}{1+j\omega \tau }+\frac{\sigma_{i}}{j\omega \varepsilon_{0}}=\varepsilon^{\prime }-j\varepsilon^{\prime \prime },$$

The dielectric properties of EG70 are described with the Davidson-Cole model [29]:

(10) $$\varepsilon^{*}=\varepsilon_{\infty }+\frac{\varepsilon_{s}-\varepsilon_{\infty }}{{\left(1+j\omega \tau \right)}^{\beta }}=\varepsilon^{\prime }-j\varepsilon^{\prime \prime },$$

where *β* is equal to 0.7582.

1-pole Cole–Cole model, Debye and Davidson–Cole model parameters for each material are given in Table 2. 

The relaxation model used to describe the dielectric properties of these materials only considered one relaxation mechanism, and that is the one occurring at microwave frequencies (1–1000 GHz range; the time constants given for different materials in Table 2. are all expressed in ps).

## 3. Results

### 3.1. Validation of The Numerical Model of the Antenna

**Figure 4:**
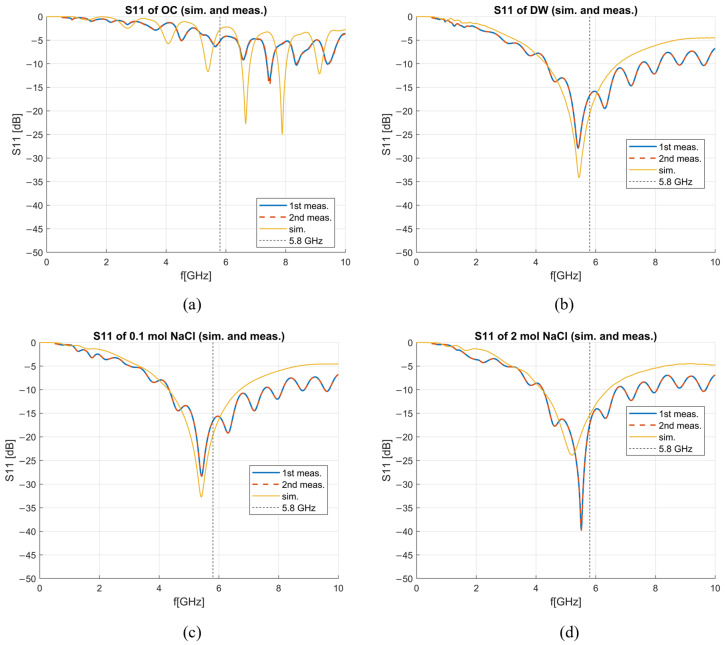
Simulated and measured reflection coefficients in dB for (**a**) open circuit, (**b**) deionized water, (**c**) 0.1 mol NaCl solution, (**d**) 2 mol NaCl solution.

Figure 4 shows the reflection coefficient of the antenna measured and simulated when left in the air (OC condition) and when inserted in deionized water, 0.1 mol NaCl solution, and 2 mol NaCl solution. Simulated results match the measurements quite well, with the exception of OC.

**Figure 5:**
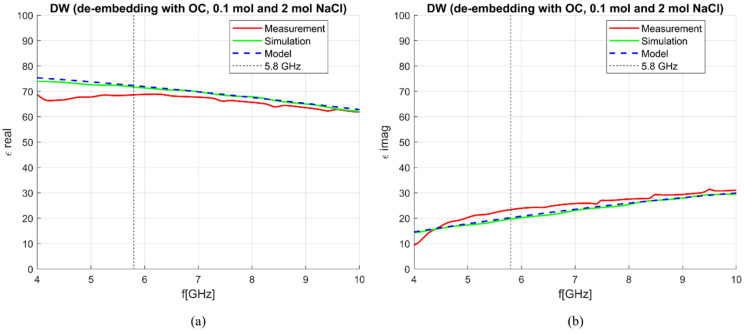
S&S de-embedding of DW with OC, 0.1 mol and 2 mol NaCl; comparison between simulations and measurements: (**a**) *ε*′; (**b**) *ε*″.

**Figure 6:**
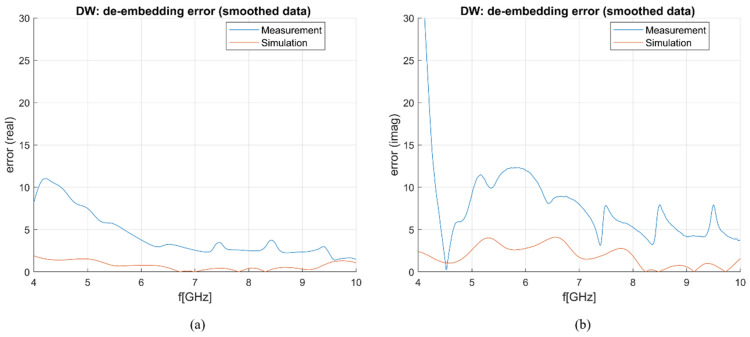
Sensitivity (de-embedding error) comparison between simulation and measurement results: (**a**) *ε*′ (“real”); (**b**) *ε*″ (“imag”).

Besides the matching of the antenna in different liquids, the ability of the antenna to measure dielectric properties needed to be verified. The S_11_ parameters obtained from both simulations and measurements were then used to de-embed properties of DW. The implemented de-embedding model was S&S, and the calibration standards were OC, 0.1 mol and 2 mol NaCl solutions. The results for DW, smoothed with a Gaussian window of size 100 [30], are compared with the theoretical model of DW dielectric properties [24] in Figure 5. The comparison of achieved antenna sensitivity based on simulated and measured results is shown in Figure 6. The frequency range in which the results are presented is 4–10 GHz due to the fact that at frequencies below 4 GHz, the sensitivity is much greater than 10%, as can be inferred by the trend in Figure 6 so that the antenna is considered unusable below such frequency for the scope at hand. The sensitivity of both simulations and measurements is below 5% for the real part of complex permittivity at frequencies above ~5.5 GHz, while at lower frequencies, the sensitivity of measurements is slightly worse as it ranges between 5 and 10% (Figure 6a). The sensitivity analysis achieved for the imaginary part of complex permittivity (Figure 6b) is better in the case of simulations with sensitivity below 5% across the entire considered frequency range (4–10 GHz), while in the case of measurements, the sensitivity is below 5% only between 4–5 GHz. Nonetheless, the overall sensitivity is good for both simulations and measurements.

### 3.2. De-Embedding Models’ Comparison

Besides the choice between two de-embedding models at disposal for calculating dielectric properties (presented in Section 2.2, calibration standards must be decided to achieve reliable results in the widest possible frequency range. In this work, a numerical analysis was performed on the capability of the antenna to de-embed the liver using different calibration liquids. In particular, if the S&S model is being used, three standards are needed. To de-embed the liver, four combinations of standards were used:

1. OC, 0.1 mol and 2 mol NaCl,
2. DW, 0.1 mol and 2 mol NaCl,
3. DW, 1 mol NaCl and 2 mol NaCl.
4. DW, 1 mol NaCl and EG70.

**Figure 7:**
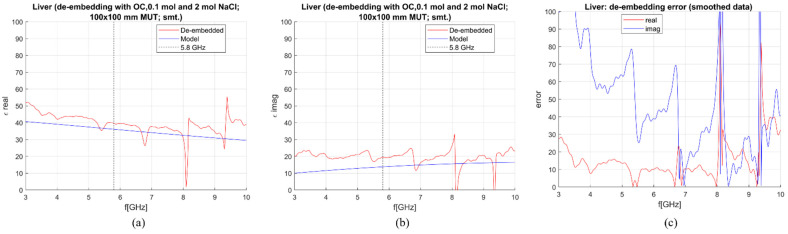
S&S de-embedding with the 1st calibration option: OC, 0.1 mol and 2 mol NaCl (smoothing applied): (**a**) Calculated vs. reference *ε*′; (**b**) calculated vs. reference *ε*″; (**c**) sensitivity (de-embedding error) for *ε*′ (“real”) and *ε*″ (“imag”).

**Figure 8:**
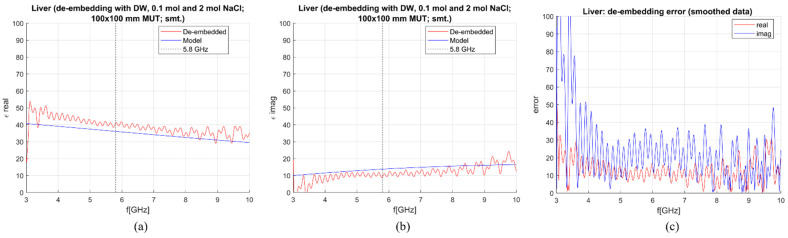
S&S de-embedding with the 2nd calibration option: DW, 0.1 mol and 2 mol NaCl (smoothing applied): (**a**) Calculated vs. reference *ε*′; (**b**) calculated vs. reference *ε*″; (**c**) sensitivity (de-embedding error) for *ε*′ (“real”) and *ε*″ (“imag”).

**Figure 9:**
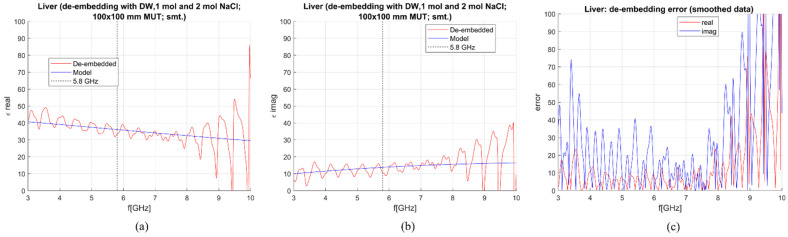
S&S de-embedding with the 3rd calibration option: DW, 0.1 mol and 2 mol NaCl (smoothing applied): (**a**) Calculated vs. reference *ε*′; (**b**) calculated vs. reference *ε*″; (**c**) sensitivity (de-embedding error) for *ε*′ (“real”) and *ε*″ (“imag”).

**Figure 10:**
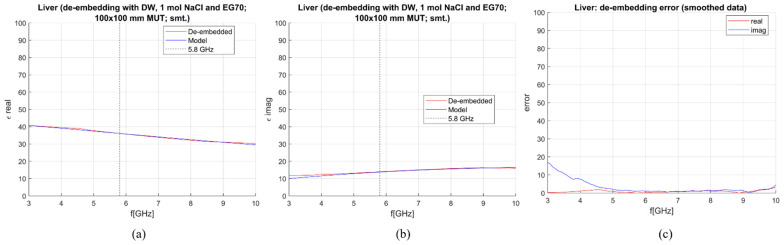
S&S de-embedding with the 4th calibration option: DW, 1 mol NaCl and EG70 (smoothing applied): (**a**) Calculated vs. reference *ε*′; (**b**) calculated vs. reference *ε*″; (**c**) sensitivity (de-embedding error) for *ε*′ (“real”) and *ε*″ (“imag”).

De-embedding of the liver and measurement sensitivity analysis were performed on simulated data in the frequency range of 0–10 GHz. The MUT was simulated as a 100 × 100 mm block with the antenna being immersed for 50 mm. In Figure 7, Figure 8, Figure 9 and Figure 10, calculated permittivity obtained with calibration options 1–4 (“De-embedded”) is plotted with the 1-pole Cole–Cole model for the liver (“Model”). In all four figures, subfigures (a) and (b) represent the real and imaginary part of complex permittivity, respectively, while (c) represents the sensitivity calculated using Equations (6) and (7). The plots consider only the frequency range between 3 and 10 GHz. This is because the sensitivity of the probe is not sufficient for measurements below ~3 GHz, regardless of the calibration option. Furthermore, shown results were smoothed out by applying the ‘smoothdata’ function in MATLAB, which gives a moving average of the elements of a vector using a fixed window length. The window length was determined heuristically [30].

The average sensitivity achieved in the frequency range of 3–10 GHz and at the operating frequency of 5.8 GHz for each calibration option is given in Table 3. De-embedded values with the first calibration option exhibit ripples in permittivity at higher frequencies. The second and third options give slightly better results in the imaginary part, but the de-embedded properties fluctuate a lot, most likely due to the too-close properties of the used calibration liquids. Finally, the de-embedding of the liver with the fourth calibration option, which uses one NaCl solution and EG70, gives the best-averaged sensitivity in the frequency range of interest. 

**Figure 11:**
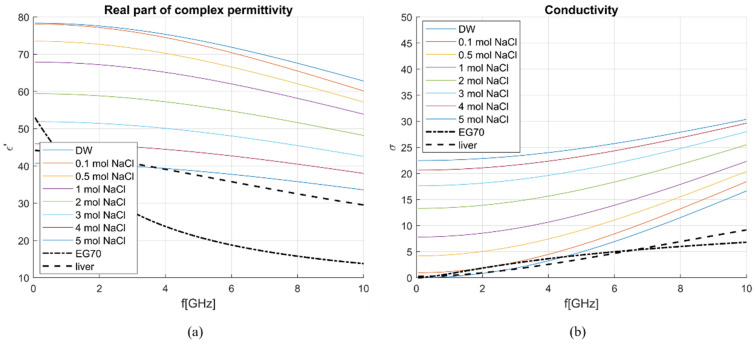
Dielectric properties of different NaCl solutions, EG70, and liver: (**a**) *ε*′ and (**b**) σ.

Figure 11 gives the overview of the dielectric properties (permittivity and conductivity) of DW [24], NaCl solutions (0.1–5 mol) [25], EG70 [26], and liver model [15]. It can be seen that the properties of solutions with a higher concentration of NaCl have lower permittivity, closer to that of the liver. Nonetheless, the higher concentration of NaCl increases the conductivity of these solutions, making them too different from liver tissue. EG70, instead, shows the real part of permittivity quite different from the permittivity of the liver, while the imaginary part is close to that of the liver. Accordingly, using both NaCl solutions and EG70 as calibration standards ensures accurate de-embedding of MUT properties.

When the M&E model is used, an additional calibration standard is needed. To de-embed the liver with the M&E model, three combinations of standards, composed from already suggested calibration liquids, were used:

1. OC, DW, 0.1 mol and 2 mol NaCl,
2. DW, EG70, 1 mol NaCl and 2 mol NaCl,
3. DW, EG70, 0.1 mol NaCl and 2 mol NaCl.

**Figure 12:**
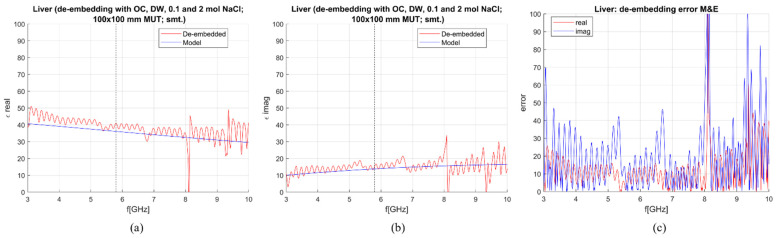
M&E de-embedding with 1st calibration option; DW, OC, 0.1 mol and 2 mol NaCl (smoothing applied): (**a**) Calculated vs. reference *ε*′; (**b**) Calculated vs. reference *ε*″; (**c**) Sensitivity (de-embedding error) for *ε*′ (“real”) and *ε*″ (“imag”).

**Figure 13:**
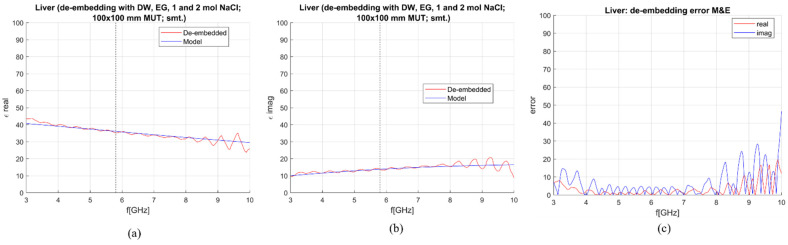
M&E de-embedding with 2nd calibration option: DW, EG70, 1 mol and 2 mol NaCl (smoothing applied): (**a**) Calculated vs. reference *ε*′; (**b**) Calculated vs. reference *ε*″; (**c**) Sensitivity (de-embedding error) for *ε*′ (“real”) and *ε*″ (“imag”).

**Figure 14:**
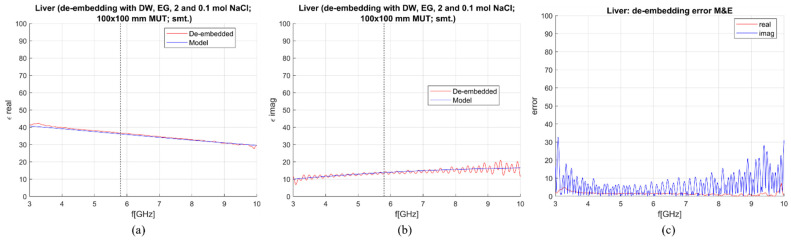
M&E de-embedding with 3rd calibration option: DW, EG70, 0.1 mol and 2 mol NaCl (smoothing applied): (**a**) Calculated vs. reference *ε*′; (**b**) calculated vs. reference *ε*″; (**c**) sensitivity (de-embedding error) for *ε*′ (“real”) and *ε*″ (“imag”).

Calculated permittivity and 1-pole Cole–Cole model of the liver are plotted against the frequency in Figure 12, Figure 13 and Figure 14. The average sensitivity achieved in the frequency range of 3–10 GHz and at 5.8 GHz for all three calibration options is given in Table 4. Comparing Table 3 and Table 4, it can be derived that with these calibration options, the M&E model does not provide a significant improvement in comparison to S&S. Therefore, it can be concluded that at this stage, the S&S model can be used for de-embedding permittivity of MUT in which the applicator is immersed in.

### 3.3. Antenna Sensitivity Analysis

#### 3.3.1. Transversal Dimension Influence

In the previous section, results were reported from 3 GHz, stating that, regardless of the calibration options, the de-embedding model yielded accurate results only at frequencies above ~3 GHz. This finding seems the opposite of the frequency limitations previously reported for the S&S de-embedding model [18]. In general, this model should be appropriate at frequencies above 500 MHz and up to 5 GHz, even though it was noticed that the upper-frequency limitation could be extended depending on the calibration [30]. The hypothesized reason behind such behavior is that in this work, an antenna is used as a sensing probe instead of an open coaxial cable; accordingly, in the case of the MUT with 100 × 100 mm transversal dimensions, at frequencies below ~3 GHz, the wave emitted by the antenna and reflected back by the MUT-air boundary influences the measured reflection coefficient. i.e., in this case, the MUT does not seem infinite to the antenna, dropping one of the main conditions that have to be satisfied to use the S&S model [18]. Therefore, when the transversal dimension decreases, it is expected that the lower frequency limitation for broadband measurements will be moved to a higher frequency. 

**Figure 15:**
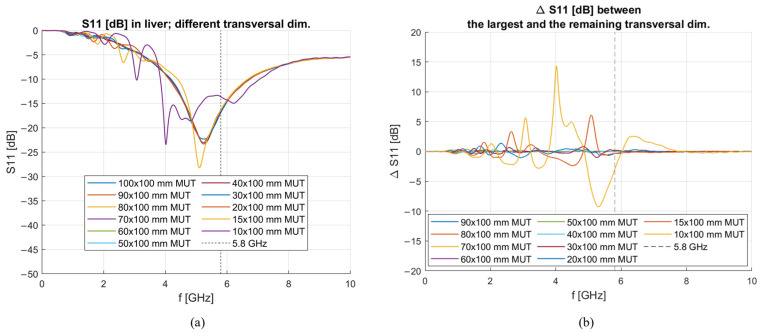
(**a**) Reflection coefficient in dB simulated in livers of different sizes (all edge lengths); (**b**) Reflection coefficient differences between simulations performed in livers of different sizes (all edge lengths).

Figure 15a gives an overview of reflection coefficients simulated when the antenna is immersed in liver blocks of different sizes. Figure 15b shows the difference between the reflection coefficient simulated in the biggest MUT and the reflections simulated in MUTs with smaller transversal dimensions. From this figure, it can be noted that these differences between reflection coefficients are more prominent below ~3 GHz, thus, confirming the incapability of the antenna to measure below such a frequency, but remain below 0.2 dB in the rest of the simulated range. The latter statement is true up to 20 mm edge. When the edge length is lower than 20 mm (15 and 10 mm), the difference becomes very big in all frequency ranges, and the matching of the antenna at 5.8 GHz is disturbed.

**Figure 16:**
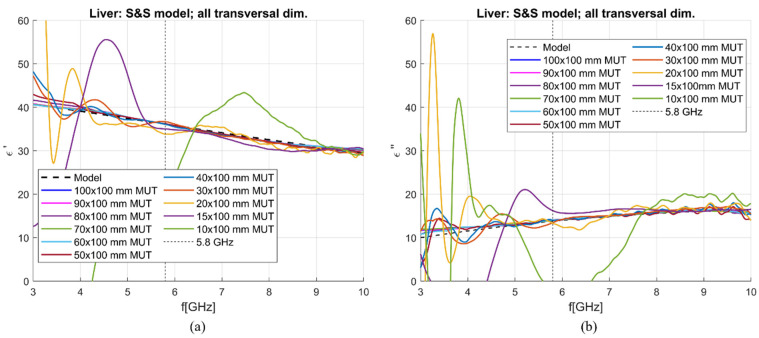
S&S de-embedding of liver simulated in different blocks: (**a**) Calculated vs. reference *ε*′ (“real”); (**b**) calculated vs. reference *ε*″(“imag”).

Figure 16 shows liver dielectric properties de-embedded using the S&S model with DW, 1 mol NaCl and EG70 for different MUT dimensions. All calibration liquids were simulated in the MUT with 100 × 100 mm transversal dimension and 100 mm longitudinal dimension, with the antenna immersed for 50 mm. Using Equations (6) and (7), the sensitivity of the applicator was calculated. Table 5 reports the sensitivity value at 5.8 GHz and as average in the 5–6 GHz band. This narrow band is used here because, decreasing the transversal dimension, the de-embedding becomes worse at frequencies higher than 3 GHz, as also evidenced by Figure 16. Achieved sensitivity at 5.8 GHz in the real part is below or around 5% for all blocks with a transversal edge length longer than 10 mm. On the other hand, the sensitivity at 5.8 GHz in the imaginary part is below or around 5% for blocks with a transversal edge longer than 15 mm.

#### 3.3.2. Longitudinal Dimension Influence

In this section, the influence of the longitudinal dimension of the MUT is studied. During the analysis, the antenna was simulated immersed into the MUT for 50 mm, while the distance between the tip of the antenna and the bottom area of the MUT was scaled from 50 mm to 2 mm (because the conical tip of the antenna is 2 mm long), with a 10 mm step. The transversal dimension of the MUT was fixed at 100 × 100 mm.

**Figure 17:**
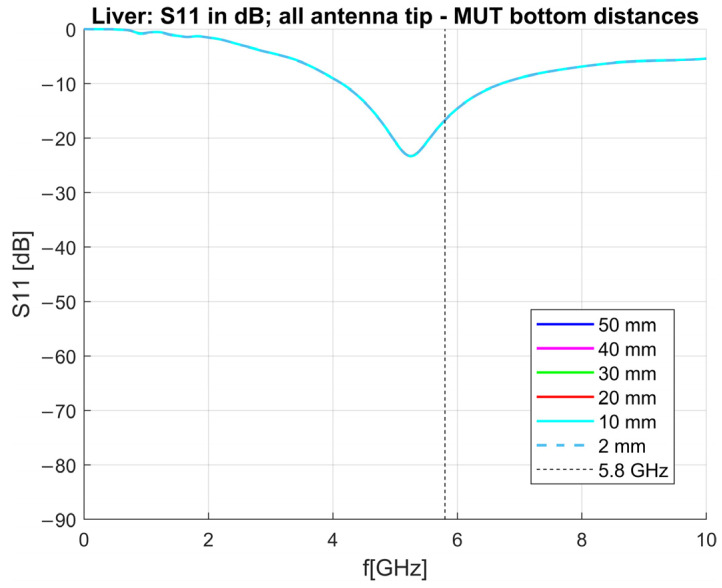
Reflection coefficient in dB simulated in livers of different antenna tip—MUT bottom distances.

The S_11_ parameters of the antenna immersed in the liver, with different distances between the antenna tip and the bottom of the MUT, are shown in Figure 17. From this figure, it can be seen that there is virtually no difference in the matching of the antenna regardless of how much distance is between the tip of the antenna and the bottom of the MUT.

**Figure 18:**
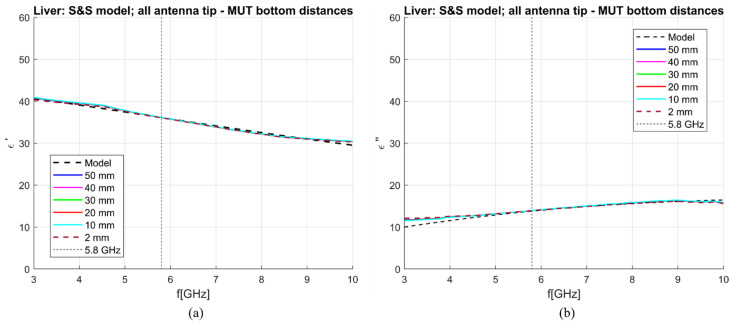
S&S de-embedding of liver simulated in different blocks: (**a**) Calculated vs. reference *ε*′ (“real”); (**b**) calculated vs. reference *ε*″(“imag”).

The S_11_ parameters obtained from simulating the antenna in different liver-filled MUTs were then used to de-embed the properties of the liver using the S&S de-embedding model. Three liquids, DW, 1 mol, and EG70 solutions, were used for calibration. These liquids were simulated in the MUT with 100 × 100 mm transversal dimension and 100 mm longitudinal dimension, with the antenna immersed for 50 mm. The obtained results are shown in Figure 18. From this figure, it is evident that regardless of the distance to the bottom of the MUT, the antenna is still able to accurately measure the dielectric properties of the surrounding liver. This result indicates that the antenna is only measuring “on the sides” and not in front of it. In this respect, it should be noted that the antenna is designed from a semirigid coaxial cable with a diameter of 1.19 mm. It was shown that open-ended coaxial probes with such small diameters also have a very small sensing region (<2 mm in the direction of the center axis of the cable) [31,32]. Accordingly, notwithstanding the open-tip design (Figure 1), the antenna is able to sense only in the radial direction.

### 3.4. Analysis of the Insertion Depth Influence

**Figure 19:**
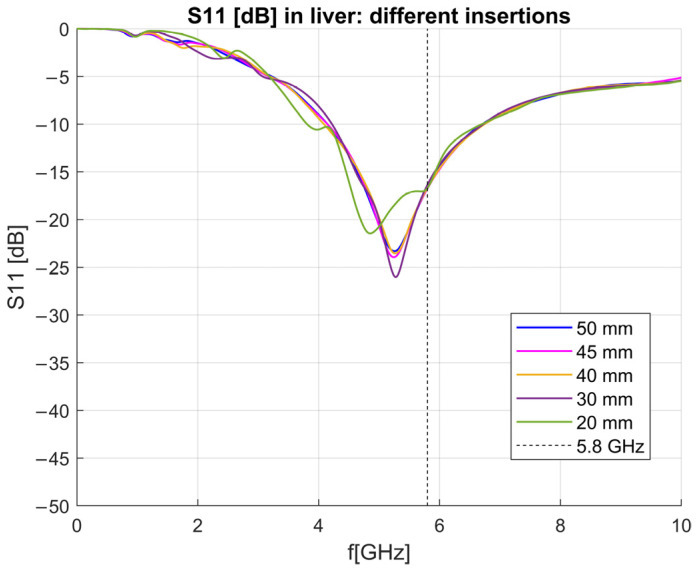
S_11_ of the antenna immersed in the liver at different lengths.

To investigate the influence of insertion depths on the antenna’s ability to measure dielectric properties, several simulations were performed. The antenna was immersed into the liver-filled simulation block of 100 × 100 × 100 mm at different depths, ranging from 50 mm to 20 mm, with a 10 mm step. The S_11_ parameters obtained from these simulations are plotted against frequency in Figure 19. It can be observed that the change in antenna immersion depth is connected to the change in the matching of the antenna. The matching of the antenna is almost the same for insertion depths 50, 45, and 40 mm, while there are prominent changes when the antenna is immersed for 30–20 mm.

**Figure 20:**
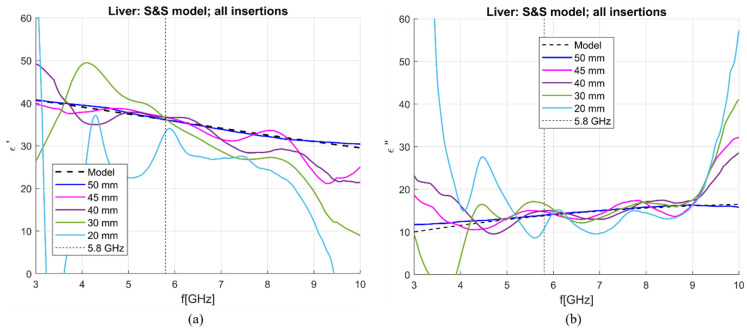
S&S de-embedding of liver simulated with different antenna immersions: (**a**) real and (**b**) imaginary part of complex permittivity.

To investigate how the antenna sensitivity behaves for different depths, the properties of the liver with the antenna immersed at different depths were de-embedded using the S&S model with DW, 1 mol NaCl and EG70 solutions. The calibration liquids were all simulated in the same MUT of 100 × 100 × 100 mm. The obtained results are shown in Figure 20, and averaged sensitivity over the 5–6 GHz frequency range is given in Table 6. Although averaged sensitivities are below 5% for the real part of permittivity with insertion depths up to 40 mm, observations of Figure 20a,b show that in that frequency range overall, the accuracy is only good at 5.8 GHz. If the entire 3–10 GHz frequency range is observed, it can be seen the results are distorted and unusable.

## 4. Discussion

In this work, the MWA antenna design proposed in [13] is realized and tested with respect to its ability to perform broadband measurements of the dielectric properties of tissues. At first, it was shown both numerically and experimentally that the antenna is well matched in different liquids such as DW, 0.1 mol and 2 mol NaCl solutions. Then, the ability of the antenna to measure dielectric properties was verified in a broad frequency range, and the obtained measurement results were in good agreement with the simulated results.

As a further step into the introduction of in situ dielectric spectroscopy in the MWA treatment, a numerical study was performed to determine the following:

- The optimal de-embedding model and calibration for reconstructing dielectric properties of MUT.
- Sensing region of the antenna.
- Immersion depth limitation.

It was found that the S&S model can be used to de-embed liver in a wide frequency band (~3–10 GHz) and that the introduction of the more complex M&E model does not give any significant improvement. A possible explanation for the achieved results, i.e., no improvement in de-embedding using a model (M&E) that takes into account the radiation resistance, even if the probe, in this case, is represented by an antenna, could be linked to the near-field interaction between the antenna and the MUT. In fact, the antenna senses the MUT, which directly surrounds it; accordingly, the greater role is likely played by the reactive near field. This electromagnetic field, as the name also evidences, is best represented by reactive elements, as the capacitances of the S&S model. With reference to the best calibration standards combination, results showed that to ensure smooth results across the frequency range, the used standards should be relatively matched at the operating frequency of the antenna. Therefore, the OC standard should be avoided in calibration as it introduces ripples in the de-embedded dielectric properties. The OC standard was used only in the 1st calibration option, which achieved the worst measurement accuracy for the imaginary part of complex permittivity. Furthermore, the prominent ripples in permittivity at higher frequencies can be correlated to the matching of the antenna in OC, as shown in Figure 4a. The OC standard is the only one among the used standards which is not relatively matched at the operating frequency of 5.8 GHz. Furthermore, the used calibration options should have properties similar to those of the MUT, as this approach ensures more accurate results. This observation is in agreement with the dielectric properties’ measurement procedure with the open-ended coaxial probe. It can also be observed that by choosing an appropriate calibration, it is possible to achieve good results at lower frequencies. The liquids used as calibration standards should preferably be biocompatible and usable for the application at hand [33]. Nonetheless, if the calibration liquid is not biocompatible, the calibration can be performed prior to the sterilization of the antenna.

From the initial analysis of the de-embedded dielectric properties based on MUT material simulated with 100 × 100 × 100 mm dimension, it was noticed that the results only become accurate at frequencies above ~3 GHz. The hypothesis behind such behavior is that the reflected wave against the boundaries of the MUT is too large, and it interferes with the resulting input admittance of the antenna. For this reason, it is expected that the lower frequency limitation for broadband measurements will move towards higher frequencies with decreasing transversal dimensions. This behavior shows that the physical size of the MUT is a limiting factor when it comes to dielectric measurements, and, therefore, it is necessary to define the antenna sensitivity at the operating frequency of 5.8 GHz and in the frequency band around it (5–6 GHz) based on the physical size of the MUT.

To investigate the latter antenna sensitivity, an analysis was performed with the antenna simulated in MUTs with different transversal and longitudinal dimensions. The transversal dimensions were changed from 100 mm to 10 mm with a 10 mm step, with an addition of a simulation in a 15 × 15 × 100 mm MUT. From this analysis, it was seen that accurate de-embedding results for the real part of complex permittivity are achieved at 5.8 GHz and in the frequency range 5–6 GHz for all simulated MUT dimensions except the smallest one with 10 × 10 × 100 mm dimensions. This is because the reflected wave becomes too great even at this frequency. This result agrees with the requirements for the size of the material when dealing with the open-ended coaxial probe technique, which requires the material to extend at least 5 mm in each direction from the probe tip [34]. Still, the sensitivity in the imaginary part is quite high in the case of the 15 × 15 × 100 mm MUT.

For the analysis of the longitudinal dimension of the MUT, the dimensions were changed so that the antenna always remains inserted for 50 mm, while the distance between the antenna tip and the bottom of the MUT was changed from 50 to 2 mm (conical tip of the antenna) with a 10 mm step. It was noticed that the antenna is not sensible to the material present beyond the tip of the antenna. Still, considering that the antenna is made from a 2.1 mm coaxial cable, there is a possibility that the material in front of the antenna tip is simply out of the sensing region of the antenna. This hypothesis is based on the previous finding about the open-ended coaxial probes constructed out of the coaxial cables with the same diameter, which have a sensing region of only 2 mm in the direction of their vertical axis [32,35].

The results related to the influence of the MUT dimension on the measurement accuracy are relevant, considering that the operating scenario will be with the antenna located in a certain position within the liver. This position is dictated by the location of the tumor, which could also be close to the boundary of the liver, i.e., close to the boundary between two different materials.

Additionally, the influence of the antenna insertion depth into the MUT on the de-embedding results was investigated. Therefore, the antenna was simulated in a liver-filled MUT at different insertion depths, from 50 to 20 mm, with a 10 mm step. It was shown that the matching of the antenna changes at 20 mm insertion depth, and this drastically affects the antenna’s ability to measure dielectric properties. It can be noted here that the antenna design features a floating sleeve to reduce reverse currents along the outer conductor of the coaxial cable and improve matching. The sleeve should make the antenna design robust with respect to the insertion depth. However, when the antenna is inserted for a depth lower than 30 mm, the final section of the sleeve is close to the boundary of the MUT, thus, influencing the results. For this reason, future work foresees an update of the antenna to look for a design able to give accurate results even at small insertion depths. Once the antenna design is improved, measurements need to be performed to compare the sensitivity analysis of physical and simulated measurements.

## 5. Conclusions

Microwave ablation has become a frequently used treatment for tumors. The success of this treatment greatly depends on precise antenna design and placement in the tissue. Different techniques, such as ultrasound and CT, are being used for guiding the antenna and monitoring the treatment. However, the ablation antenna itself has the potential to ensure proper placement in the tumorous tissue and for monitoring the treatment in real-time. For this reason, the measurement sensitivity of the open-ended coaxial slot antenna designed to operate in liver tissue at 5.8 GHz was investigated in the 0–10 GHz frequency range and at the operating frequency. The ability of the antenna to measure the dielectric properties of the targeted tissue greatly depends on the calibration setup used. This research showed that the antenna should be matched in the calibration liquids and that the dielectric properties of at least one liquid should be close to that of the targeted tissue. Furthermore, the influence of the transversal and longitudinal dimensions of the MUT on the measurement sensitivity was studied. While the longitudinal dimension (the distance between the tip of the antenna and the bottom of the MUT) does not influence the measurement results, the transversal dimension showed to be a limiting parameter for broadband dielectric spectroscopy. Generally, the broadband measurement accuracy in the case of the 100 × 100 mm MUT is limited to 3–10 GHz. The decrease in the transversal dimension causes the measurement accuracy to deteriorate at lower frequencies (<5 GHz). However, the minimum transversal dimension at which the antenna is able to measure dielectric properties at the operating frequency is as small as 15 × 15 mm. The analysis of the antenna insertion depth influence on the measurement results showed that this antenna design could give accurate results when the antenna is inserted up to 40 mm. At lower insertion depths, the measurement accuracy becomes inadequate.

## Figures and Tables

**Table 1:** Design parameter overview.

Design Parameter	Parameter Value [mm]
**Antenna: structure**	
$h_{\mathrm{tip}}$	2
$d_{\mathrm{slot}}$	7.7
$l_{\mathrm{slot}}$	1.75
$d_{\mathrm{sleeve}}$	0.55
$l_{\mathrm{sleeve}}$	5.28
$l_{\mathrm{cable}}$	100
$l_{\mathrm{insertion}}$	50
**Antenna: cross-section**	
$r_{\mathrm{inner}\;\mathrm{conductor}}$	0.15
$r_{\mathrm{dielectric}}$	0.485
$r_{\mathrm{outer}\;\mathrm{conductor}}$	0.595
$r_{\mathrm{PTFE}\;\mathrm{layer}\;}$	0.795
$r_{\mathrm{sleeve}}$	1.095
$r_{\mathrm{outer}\;\mathrm{tubing}}$	1.295
**MUT block (cube)**	
Cube edge	100

**Table 2:** 1-pole Cole–Cole model parameters of simulated materials.

Material	$\varepsilon_{S}$	$\varepsilon_{\infty }$	τ/ps	$\sigma_{i}(S/m)$	$\varepsilon^{*}\;\mathrm{at}\;5.8\;\mathrm{GHz}$
Liver [15]	44.32	5.32	11.55	0.25	36.115–j13.799
DW at 25 °C [24]	78.36	5.2	8.27	/ *	72.268–j20.213
0.1 mol NaCl (at 20 °C) [25]	78.1	5.22	9.1	0.96	70.879–j22.071
1 mol NaCl (at 20 °C) [21]	67.9	5.22	8.53	7.81	62.377–j41.971
2 mol NaCl (at 20 °C) [21]	59.4	5.22	8.13	13.29	55.028–j55.944
EG70 (at 25 °C) [26]	53.96	3.99	58.34	/ *	19.175–j15.080

* Debye and Davidson–Cole relaxation models without ionic conductivity term.

**Table 3:** Averaged sensitivity in the 3–10 GHz range and at 5.8 GHz for S&S de-embedding with different calibration options.

	Measurement Sensitivity			
	3–10 GHz Range		@5.8 GHz	
Calibration Option	$\Delta_{real}\;\left[\%\right]$	$\Delta_{imag}\;\left[\%\right]$	$\Delta_{real}\;\left[\%\right]$	$\Delta_{imag}\;\left[\%\right]$
OC, 0.1 mol and 2 mol NaCl	13.15	40.59	6.61	32.30
DW, 0.1 mol and 2 mol NaCl	12.95	26.11	13.52	33.13
DW, 1 mol and 2 mol NaCl	15.44	33.84	7.57	23.22
DW, 1 mol NaCl and EG70	0.85	3.09	0.07	1.09

**Table 4:** Averaged sensitivity in the 3–10 GHz range and at the operating frequency for M&E de-embedding with different calibration options.

	Measurement Sensitivity			
	In the 3–10 GHz Range		At 5.8 GHz	
Calibration Option	$\Delta_{real}\;\left[\%\right]$	$\Delta_{imag}\;\left[\%\right]$	$\Delta_{real}\;\left[\%\right]$	$\Delta_{imag}\;\left[\%\right]$
OC, DW, 0.1 mol and 2 mol NaCl	12.75	20.70	13.35	11.52
DW, EG70, 1 mol and 2 mol NaCl	3.28	6.61	2.46	0.14
DW, EG70, 0.1 mol and 2 mol NaCl	1.48	6.57	1.42	3.20

**Table 5:** Average sensitivity in the 5–6 GHz range and at 5.8 GHz for different sizes of MUT.

	Measurement Sensitivity			
	In the 5–6 GHz Range		At 5.8 GHz	
Liver Block Size [mm×mm]	$\Delta_{real}\;\left[\%\right]$	$\Delta_{imag}\;\left[\%\right]$	$\Delta_{real}\;\left[\%\right]$	$\Delta_{imag}\;\left[\%\right]$
100 × 100	0.29	1.33	0.07	1.09
90 × 100	0.30	1.26	0.04	1.02
80 × 100	0.08	1.85	0.29	1.42
70 × 100	0.28	1.34	0.06	1.02
60 × 100	0.28	1.46	0.10	1.56
50 × 100	0.39	1.38	0.26	1.39
40 × 100	0.33	1.48	0.10	1.50
30 × 100	2.13	4.74	1.56	2.55
20 × 100	4.28	4.48	6.36	3.46
15 × 100	6.17	37.37	3.04	17.55
10 × 100	58.22	79.88	45.76	121.88

**Table 6:** Average sensitivity in the 5–6 GHz range and at 5.8 GHz for different immersion lengths.

	Measurement Sensitivity			
	In the 5–6 GHz Range		At 5.8 GHz	
Antenna Immersion [mm]	$\Delta_{real}\;\left[\%\right]$	$\Delta_{imag}\;\left[\%\right]$	$\Delta_{real}\;\left[\%\right]$	$\Delta_{imag}\;\left[\%\right]$
50	0.29	1.33	0.07	1.09
45	2.00	7.89	1.61	7.06
40	1.55	7.33	1.66	8.64
30	5.57	17.21	0.66	20.08
20	24.98	20.15	7.81	21.97

## Data Availability

The data presented in this study are available on request from the corresponding author.
